# Use of tamoxifen and aromatase inhibitors in a large population-based cohort of women with breast cancer

**DOI:** 10.1038/bjc.2011.140

**Published:** 2011-04-26

**Authors:** L Huiart, S Dell'Aniello, S Suissa

**Affiliations:** 1 Department of Cancer Genetics, Prevention and Screening, Institut Paoli-Calmettes, 232 Bd Saint-Marguerite, Marseille, France; 2 Inserm, UMR912, SES4, Institut Paoli-Calmettes, Marseille, France; 3 Centre for Clinical Epidemiology and Community Studies, Sir Mortimer B Davis Jewish General Hospital, Montreal, Quebec, Canada; 4 Department of Epidemiology, Biostatistics and Occupational Health, McGill University, Montreal, Quebec, Canada

**Keywords:** breast cancer, pharmacoepidemiology, tamoxifen, aromatase inhibitors, compliance, persistence

## Abstract

**Background:**

Non-compliance with oral treatment in oncology is an emerging health issue. For breast cancer (BC) patients, few data are available on compliance and persistence to tamoxifen in younger women and to aromatase inhibitors (AIs) as compared with tamoxifen in older women.

**Methods:**

We constituted a cohort of 13 479 women with BC who received at least one prescription of tamoxifen or AI between 1998 and 2008, in the United Kingdom General Practice Research Database. Days covered by medication and treatment discontinuation were studied. Time to treatment discontinuation was calculated using Kaplan–Meier estimates.

**Results:**

Overall, 18.9% (95% CI: 15.1–23.0) of women on AIs as compared with 31.0% (95% CI: 29.6–32.2) of women on tamoxifen had discontinued their treatments within the first 5 years (*P*<0.001). This rate raised to 50.7% (95% CI: 43.0–57.9) among the 416 women under 40 years receiving tamoxifen as initial hormonal therapy. Among older women, treatment discontinuation was less frequent for AIs as compared with tamoxifen (*P*<0.001). Among women on AI therapy, 14% of them (*n*=374) had switched treatments.

**Conclusion:**

Among older women, the real-life patterns of use of AI show high rates of compliance. In younger women, tamoxifen is prematurely discontinued for half of patients.

In the field of oncology, the use of oral therapy is increasing and treatment compliance issues are of growing interest (Partridge *et al*, 2002; Ruddy *et al*, 2009). Oral adjuvant hormonal therapy in hormonal-responsive early breast cancer (BC) reduces the risk of recurrence and increases survival rates (Early Breast Cancer Trialists’ Collaborative Group (EBCTCG), 2005). For the last 20 years, a 5-year tamoxifen treatment was the standard therapy for all women diagnosed with hormone-sensitive BC. More recently, aromatase inhibitors (AIs) were shown to further reduce the risk of recurrence or death from BC as compared with tamoxifen in post-menopausal women (Crivellari *et al*, 2008; Forbes *et al*, 2008) and constitute an alternative option now recommended for the adjuvant treatment of early BC (National Institute for Health and Clinical Excellence, 2006; Goldhirsch *et al*, 2009; Burstein *et al*, 2010).

Non-compliance and early discontinuation of hormonal treatments are likely to affect treatment efficacy in BC patients (Early Breast Cancer Trialists’ Collaborative Group (EBCTCG), 2005; McCowan *et al*, 2008; Dezentje *et al*, 2009). In previously published observational studies, depending on the methodology used, discontinuation rates for tamoxifen ranged from 17% at 2 years to 49% at 5 years (Demissie *et al*, 2001; Partridge *et al*, 2003; Fink *et al*, 2004; Lash *et al*, 2006; Barron *et al*, 2007; Kahn *et al*, 2007; Owusu *et al*, 2008; Ziller *et al*, 2009; Hershman *et al*, 2010) and from 21 to 51% at a 3-year maximal follow-up for AIs (Partridge *et al*, 2008; Ziller *et al*, 2009; Hershman *et al*, 2010). Comparison of non-persistence between tamoxifen and AIs is difficult as the majority of past studies have often used different methodologies rendering external comparison inappropriate.

Tamoxifen and AIs present different side-effect profiles that may in turn affect the patient's compliance with the treatment differently outside controlled clinical trials. Specific functional side effects of AIs such as arthralgia, observed in routine check-ups, are likely to further reduce compliance. The superiority of the efficacy of AIs over tamoxifen observed in clinical trials, where compliance is likely to be optimal (Osterberg and Blaschke, 2005), may be diminished in real life owing to differential non-compliance rates. Studying further the difference in compliance between tamoxifen and AIs is therefore crucial.

Few data are available on compliance with and persistence to tamoxifen in younger women. Indeed, most studies focused on older women and excluded premenopausal women. Only four studies included women with a large age range (Partridge *et al*, 2003; Barron *et al*, 2007; Kahn *et al*, 2007; Hershman *et al*, 2010). These four studies found younger age to be associated with lower compliance or higher discontinuation rates. However, they did not provide detailed data for this age group and relied on self-reports of compliance or on databases covering specific subsamples of the general population. Therefore to date, no study on younger women has provided detailed data on tamoxifen use in a population-based setting.

Our objective was therefore to study patterns of use of tamoxifen and AIs in an observational population-based cohort of women with BC. We secondly focused on two specific aspects: first, tamoxifen use in younger women with BC; second, AI use as compared with tamoxifen use in older women.

## Methods

### Source of data

The United Kingdom (UK) General Practice Research Database (GPRD) constituted the primary source of data. It comprises data on over 4.4 million currently active individuals (approximately 7% of the English population) collected prospectively from 1987 to present. Data collected to date comprise over 55 million person years of research quality data. As the UK National Health Service provides universal coverage, the database is population based, all segments of the population being represented. The medical practices participating in the GPRD show a geographic distribution representative of the UK population. Age and sex distributions of patients in the GPRD reflect those reported by the National Population Census (Garcia Rodriguez *et al*, 2002).

In the UK, GPs are the primary care givers for all patients in the National Health Service and therefore all consultants are required to inform the referring GP on clinical events and medical diagnoses whenever a patient is seen in hospital or by an outpatient specialist.

The database records a large amount of medical information including comprehensive records of prescriptions written, all clinically medical diagnoses both in-patient and outpatient, and referrals to hospitals. Prescriptions supplied by GPRD physicians are automatically recorded in the database. The recorded information on drug exposures and diagnoses has been validated (Lawrenson *et al*, 1999, 2000; Jick *et al*, 2003). More specifically, the GPRD has been shown to provide BC incidence closely similar to the national registration data (Kaye *et al*, 2000).

The study protocol was approved by the Scientific and Ethical Advisory Group of the GPRD.

### Study population

#### Cohort definition.

We constituted a cohort of women diagnosed with BC and who received at least one prescription of tamoxifen, anastrozole, letrozole, or exemestane between 1 January 1998 and 30 June 2008. Breast cancer was identified using a code for breast surgery combined with a diagnostic code of invasive cancer. All diagnoses were identified using a list of codes for diagnosis and procedures available in the GPRD relying on the READ classification. Medications from the recorded prescriptions are identified using the coded drug dictionary based on the UK Prescription Pricing Authority Dictionary.

Cohort entry was defined as the date of first prescription of tamoxifen/AI. The maximum delay between the diagnostic code and the first prescription was 1 year to increase the likelihood that the treatment was prescribed as an adjuvant treatment for primary BC. We excluded patients who had a previous history of BC. We also excluded women who were metastatic at diagnosis or within the first 6 months after diagnosis. Patients who were prescribed simultaneously tamoxifen and AI and patients who had less than a year of data before cohort entry were also excluded.

All patients were followed from cohort entry to the end of treatment (theoretically 5 years), or until death from any cause, BC recurrence or contralateral BC, thrombo-embolic event, or endometrial cancer, whichever came first. If a patient switched between tamoxifen and AI or between two AIs during the study period, follow-up was censored at the time of the switch to calculate the compliance and persistence to the first drug.

Subcohorts were constituted according to age and date of diagnosis to account for the changes over time in clinical guidelines. Compliance and persistence were studied separately for women under 40 years of age and women 50 years and over at the time of diagnosis. For the younger group, tamoxifen was the only treatment studied as it remains the main therapeutic option for hormonal treatment in this mostly pre-menopausal subgroup of patients (Tam 40). For the latter group, owing to the recent recommendations on the use of AIs as adjuvant hormonal treatment in post-menopausal patients, these women were stratified according to the date of diagnosis to create an historical comparison group of tamoxifen users. This reference group was defined as having a diagnostic date before 2000 (Tam 50), when tamoxifen was generally prescribed for a 5-year period. Compliance and persistence to medication in this reference group was compared with the compliance and persistence to AIs among women diagnosed after 2006 (AI 50). Indeed, anastrozole and letrozole were licensed as adjuvant treatments for early BC in post-menopausal women in 2004 and 2005, respectively, in the UK, and UK clinical guidelines were updated in 2006 (National Institute for Health and Clinical Excellence, 2006).

#### Definition of measures of medication compliance and persistence.

Compliance was defined as the numbers of days covered by medication divided by the number of days between cohort entry and end of study participation. This proportion corresponds to the medication possession ratio (MPR) (Andrade *et al*, 2006; Cramer *et al*, 2008). For each woman, number of days covered by medication was calculated from the number of tablets prescribed combined with dosing instruction. From this information, we calculated the duration of the prescription. Durations of overlapping prescriptions were not added. A conventional cutoff of 80% or more days covered was used to calculate the proportion of women who where compliant with treatment (Fisher *et al*, 1989; Love, 1990; Waterhouse *et al*, 1993; Partridge *et al*, 2008). Women whose MPR was less than 80% were considered as non-compliant. We studied non-persistence to treatment defined as the first treatment discontinuation longer than 3 months. A gap of 3 months or more in medication coverage identified treatment discontinuation.

All treatments were handled similarly to limit differential misclassification of exposure.

### Statistical analysis

Descriptive statistics were computed for continuous data (mean±standard deviation (s.d.)) and categorical data (sample size and percentage). Time to treatment discontinuation was calculated using Kaplan–Meier estimates to account for censored data. Patients follow-up was censored at 5 years (the theoretical duration of treatment), or at the date of death, BC recurrence or contralateral BC, thrombo-embolic event, endometrial cancer, or switch to a different antihormonal treatment, whichever came first. Results are expressed as cumulative probabilities of treatment discontinuation with 95% confidence intervals (CIs). Kaplan–Meier estimates of treatment discontinuation of the different subgroups were compared using the log-rank test.

All analyses were conducted with SAS version 9.1 (SAS Institute, Cary, NC, USA).

## Results

**Figure 1:**
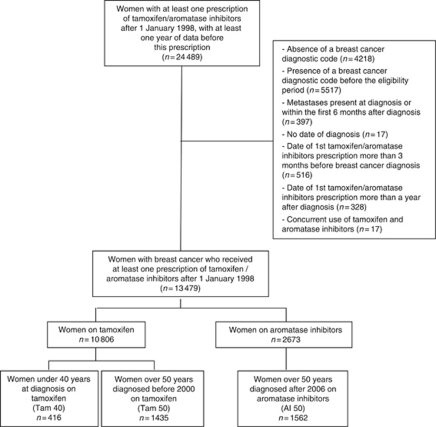
Study flow chart.

In the GPRD, we identified 24 489 women who received a first prescription of tamoxifen, anastrozole, letrozole or exemestane after 1 January 1998, who also had at least 1 year of data before this prescription in the database. We excluded 4218 women owing to the absence of a BC diagnostic code in their records, 5517 women because BC was diagnosed before the study period, 397 women because of metastases before or within the first 6 months after diagnosis and 17 had no date of diagnosis. We further excluded 516 women who received their first prescription more than 3 months before BC diagnosis, 328 who received their treatment more than a year after diagnosis and 17 women who were prescribed two drugs concomitantly (Figure 1). We therefore constituted a cohort of 13 479 women who received tamoxifen (*n*=10 806) or one of the AIs (*n*=2673) following the diagnosis of BC. Mean age at cohort entry was 62 years (s.d.=14.0) in the tamoxifen group and 70.8 (s.d.=12.4) in the AI group (Table 1).

We identified 416 women under 40 receiving tamoxifen as initial hormonal therapy (Tam 40), 1435 women over 50 diagnosed with BC before 2000 and receiving tamoxifen (Tam 50) and 1562 women over 50 diagnosed after 2006 and receiving AI (AI 50).

**Figure 2:**
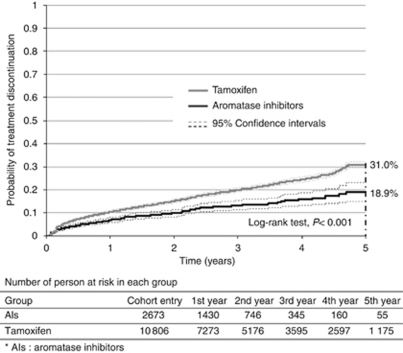
Time to treatment discontinuation – medication gap longer than 3 months.

In the entire cohort, treatment discontinuation reached 29.8% of patients at 5 years. This rate ranged from 18.9% (95% CI: 15.1–23.0) for AIs users to 31.0% (95% CI: 29.6–32.2) for tamoxifen users (*P*<0.001) (Figure 2).

**Figure 3:**
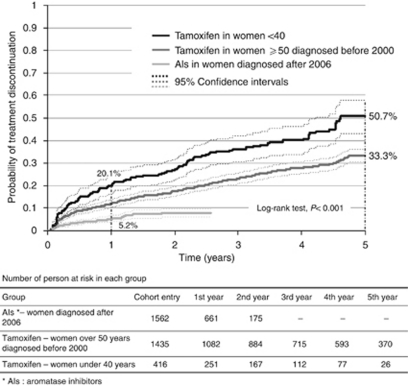
Time to treatment discontinuation – medication gap longer than 3 months.

In the three subcohorts, rates of the treatment discontinuation in the first year ranged from 5.2% (95% CI: 4.0–6.6) to 20.1% (95% CI: 16.2–24.2) in the AI 50 and in the Tam 40 groups, respectively (Figure 3). At 5 years, more than half of the women (50.7% 95% CI: 43.0–57.9) in the Tam 40 group had discontinued their treatment. Among women over 50, treatment discontinuation was less frequent for AIs as compared with tamoxifen (*P*<0.001).

Table 2 describes non-compliance, that is, MPR lower than 80% for patients receiving at least one prescription within that year. In the first year of treatment, non-compliance ranged from 9.5% in the AI 50 group to 23.6% in the Tam 40 group. Non-compliance reached 51.8% at 5 years in the latter group.

In the AI 50 group, 9.6% of women (150 out of 1562) had switched treatment by June 2008, corresponding to last data available for our study. Half of them switched from one AI to another AI, and the other half switched from AI to tamoxifen. Switches occurred within the first year of treatment in 76% of cases. Among women over 50 years of age who started a tamoxifen therapy *after* 2000, 31% switched to AI over the course of the study. Of these women, 12% switched within the first year of treatment (11.1 and 13.7% for women diagnosed in 2000–2004 and after 2005, respectively). A switch within the first year indicates a change in treatment not in accordance with medical guidelines, which recommend continuing tamoxifen treatment for a minimum of 2–3 years. This may indicate a switch for other reasons such as side effects.

## Discussion

Among older women, the real-life patterns of use of AI show high rates of persistence to treatment. As compared with tamoxifen, AI therapy appears to be less frequently discontinued. In younger women, tamoxifen is prematurely interrupted for half of patients.

In clinical trials, compliance is likely to be optimal owing to the close monitoring of patients and the volunteer nature of such studies (Osterberg and Blaschke, 2005). Indeed, in the three largest trials on adjuvant tamoxifen for BC, about 20% of women discontinued their treatment prematurely (2001). In the ATAC trial, 88% of women allocated to AIs as compared with 87% on tamoxifen reported to be compliant with their treatment at 5 years (Forbes *et al*, 2008). Compliance rates in observational studies such as ours are expected to be lower than those reported in trials, and indeed our results are closer to those of previously published observational studies. Partridge *et al* (2008) reported for the first time data on AI compliance in a large commercial database. The mean compliance with anastrozole ranged from 82 to 88% in the first 12 months of treatment, and from 62 to 79% in the third year. Our results, taking into account that Partridge *et al* (2008) were focusing on prescription claims, whereas we are studying the prescription issued by the physician, are consistent.

However, our results comparing tamoxifen to AI use in older women contrast with these from the only comparative study available to date (Ziller *et al*, 2009). This German retrospective study on women initiating a treatment in 2004–2005 reported, in a small sample of women (*n*=89), a lower compliance with anastrozole (80 *vs* 69%). In the study time period, anastrozole was not a standard treatment according to the German treatment guidelines, although already largely prescribed. In this study, women who received anastrozole were older (65 years old on average for tamoxifen *vs* 72 for anastrozole) and had different tumour characteristics as compared with those receiving tamoxifen (e.g., 20% of *in situ* tumours for tamoxifen as compared with 3% for anastrozole) (i.e., indication bias). The different characteristics of women in the two groups may translate into different compliance rates. Oldest age group has been showed to be associated with lower compliance rates (Partridge *et al*, 2003; Barron *et al*, 2007; Kahn *et al*, 2007; Owusu *et al*, 2008). As tamoxifen is nowadays considered a less efficient adjuvant therapy to lower the risk of BC recurrence, it is in most cases only prescribed owing to personal characteristics of the patient (indication bias). To avoid this bias, compliance with AIs should be compared to compliance with tamoxifen when tamoxifen was the only therapeutic option. This is the reason why we used an historical cohort for the tamoxifen reference group when this latter treatment was the reference treatment.

Negative beliefs about treatment are known to decrease compliance in numerous diseases (Horne and Weinman, 1999). A similar association was found for tamoxifen in BC (Fink *et al*, 2004; Lash *et al*, 2006). Little is known on patients’ beliefs on AIs. However, as physicians were prompt to adopt AIs in replacement of tamoxifen even before publications of guidelines (Buzdar and Macahilig, 2005; Chlebowski, 2008), one can speculate that physicians were confident in the efficacy of a newer treatment and conveyed this message to patients. This may be a possible explanation for the lower rates of treatment discontinuation among women on AI, observed in our study. We need further studies and data to support this hypothesis. An alternative or complementary explanation may be a better treatment tolerance in the AI group as compared with tamoxifen as previously reported in the ATAC trial (Buzdar *et al*, 2006).

Previous observational studies on Tam have identified younger age as a factor associated with lower compliance (Partridge *et al*, 2003; Barron *et al*, 2007; Kahn *et al*, 2007; Hershman *et al*, 2010). One of these studies focused on self-report of compliance, likely to provide overestimates of compliance (Kahn *et al*, 2007). The three other studies reported prescription refill rates as estimates of compliance rates; however, they used either commercial database (Hershman *et al*, 2010) or databases comprising mostly socially disadvantaged women (Partridge *et al*, 2003; Barron *et al*, 2007). However, in the subgroup of younger patients, no specific description of the rates of compliance and patterns of treatment discontinuation has been provided. The rapid decrease in use of tamoxifen that we observed is of particular concern in this subgroup of women. They present poorer outcomes of BC as compared with older women (Adami *et al*, 1986; Chung *et al*, 1996; Gnerlich *et al*, 2009). There is no alternative recommended hormonal treatment available and as it has been established that a 1- or 2-year treatment is less effective than a full 5-year course (1998). The high rates of non-persistence contrast with the study of Thewes *et al* (2005) reporting, using patient preference interviews, that modest gains in survival were sufficient for women under 40 with BC to find worthwhile a 5-year tamoxifen treatment. The reasons for early treatment discontinuation are largely unknown in younger women. However, several specificity of this subgroup may have a role in the difference of compliance observed. Younger women face specific consequences of systemic treatment including menopausal symptoms, sexual dysfunction, and fertility issues (Fallowfield *et al*, 1999; Pellegrini *et al*, 2009), and they also suffer from higher psychosocial distress and lower quality of life after BC as compared with older women (Mor *et al*, 1994; Ganz *et al*, 2003).

Our study has several strengths. First, it is population based as opposed to studies using commercial health programme databases. These latter sources of data provide results that may be systematically biased because of the exclusion of different subgroups of the general population depending on the programme eligibility. Access to medical care and cost of treatment is likely to impact compliance. In the UK, National Health Services provide universal coverage. During the time period of the study, treatments were dispensed with a fixed prescription charge per item. Exemption of prescription charges are based on patient characteristics (e.g., over 60 years of age). Use of treatment is measured in our study with no differential cost issues between tamoxifen and AIs. The difference observed between the two treatments is therefore independent of cost issues. Second, the validity of the information recorded in our source of data, the GPRD, has been largely studied and shown to be high. In terms of the recorded information on drug exposures and diagnoses, the GPRD has been validated (Lawrenson *et al*, 1999, 2000; Jick *et al*, 2003). More specifically, this database provides BC incidence closely similar to that of the national registration data (Kaye *et al*, 2000). Third, the size of the database enabled the selection of large samples even for our smaller group, BC in younger women. Fourth, we are studying prescriptions written by physicians, an objective measure of exposure, free of recall bias because of the prospective nature of data collection in the GPRD.

A limitation of our study is that we do not have access to exact data on patient compliance. Prescriptions written by physician overestimate what patients actually ingest. However, although methodologies to evaluate compliance are numerous, they all have advantages and disadvantages and none is considered as a gold standard (Osterberg and Blaschke, 2005). Rate of prescription refills is an objective measure and has been shown in countries with universal health coverage to provide accurate measures of overall compliance (Lau *et al*, 1997). We use a proxy measure of prescription refills as we have access to prescription issued by the GP. Moreover, the clinical significance of our results is not modified by the fact that we underestimate treatment discontinuation rates. At least half of women stop their treatment prematurely, a proportion large enough to stress the need for intervention to improve compliance with and maintenance of treatment. Third, while tamoxifen and AI use may be overestimated, this overestimation affects both groups similarly and therefore the magnitude of the difference between younger and older women and between tamoxifen and AI is not likely to be modified.

To our knowledge, our study is the first large observational study to report comparative data on the use of AIs as compared to tamoxifen and to provide detailed information on real-life use of tamoxifen in younger women.

## Figures and Tables

**Table 1:** Characteristics of study population

**Characteristics**	**Tamoxifen**	**AIs**	**Tamoxifen in women under 40 years**	**Tamoxifen in women over 50 years diagnosed before 2000**	**AIs in women diagnosed after 2006**
Number of patients	10 806	2673	416	1435	1562
Mean age at cohort entry (s.d.)	62.0 (14.0)	70.8 (12.4)	35.9 (3.3)	67.4 (12.0)	69.7 (12.0)
Mean follow-up in years (s.d.)	2.7 (1.8)	1.6 (1.3)	2.8 (1.7)	3.5 (1.8)	1.0 (0.7)
Switch between treatments (%)	2827 (26.2)	374 (14.0)	45 (10.8)	194 (13.5)	150 (9.6)
BC recurrence/or contralateral BC (%)	547 (5.1)	117 (4.4)	66 (15.9)	114 (7.9)	27 (1.7)
Death (%)	652 (6.0)	276 (10.3)	8 (1.9)	165 (11.5)	77 (4.9)
Thrombo-embolic event (%)	370 (3.42)	84 (3.14)	4 (0.9)	91 (6.3)	32 (2.1)
Endometrial cancer (%)	20 (0.19)	3 (0.11)	1 (0.24)	2 (0.14)	1 (0.06)
NSAIDs or ASA use	3443 (31.9)	1090 (40.8)	88 (21.2)	489 (34.1)	590 (37.8)

Abbreviations: AIs=aromatase inhibitors; ASA=acetylsalicylic acid; BC=breast cancer; NSAIDs=non-steroidal anti-inflammatory drugs; s.d.=standard deviation.

**Table 2:** Treatment coverage according to medication, age, and duration of treatment

	**1st year**	**2nd year**	**3rd year**	**4th year**	**5th year**
*AIs in women diagnosed after 2006*					
*n*	1562	702	185	—	—
<80% of days covered	149 (9.5%)	90 (12.8%)	33 (17.8%)	—	—
					
*Tamoxifen in women over 50 years diagnosed before 2000*					
*n*	1435	1230	1057	914	801
<80% of days covered	217 (15.1%)	210 (17.1%)	212 (20.1%)	198 (21.7%)	220 (27.5%)
					
*Tamoxifen in women under 40 years*					
*n*	416	321	235	184	139
<80% of days covered	98 (23.6%)	101 (31.5%)	81 (34.5%)	76 (41.3%)	72 (51.8%)

Abbreviation: AIs=aromatase inhibitors.
